# Decision tree for selection of suitable cultivation parameters for mammalian cell culture processes

**DOI:** 10.1186/1753-6561-9-S9-P45

**Published:** 2015-12-14

**Authors:** Ralf Pörtner, Simon Kern, Dieter Eibl

**Affiliations:** 1 Institute of Bioprocess and Biosystems Engineering, Hamburg University of Technology, Hamburg, D-21073, Germany; 2 Institute of Biotechnology, Biochemical Engineering and Cell Cultivation Technique, Zurich University of Applied Sciences, 8820 Wädenswil, Switzerland

## Background

Development of bioprocesses for mammalian cells has to deal with different bioreactor types and scales. Bio-reactors might be intended for seed train and production, research, process development, validation or transfer purposes. During these activities, not only the problem of up- and downscaling might lead to failure of repro-ducibility, but also the use of different bioreactor geometries and operation conditions. In such cases, the criteria for bioreactor design and process transfer should be re-evaluated in order to avoid an erroneous transfer of cultivation parameters.

## Concept

For selection of process conditions several questions can be asked:

• Type and scale of the intended cultivation system

• Which data are required (cell specific parameters, specific data for the cultivation system)?

• Are appropriate data e.g. for cell growth, substrate uptake, medium composition available?

• For which cultivation systems have these data been determined?

• Are data on power input, mixing time, oxygen transfer etc. available?

• Which methods can be used to determine or estimate the above mentioned parameters?

**Figure 1:**
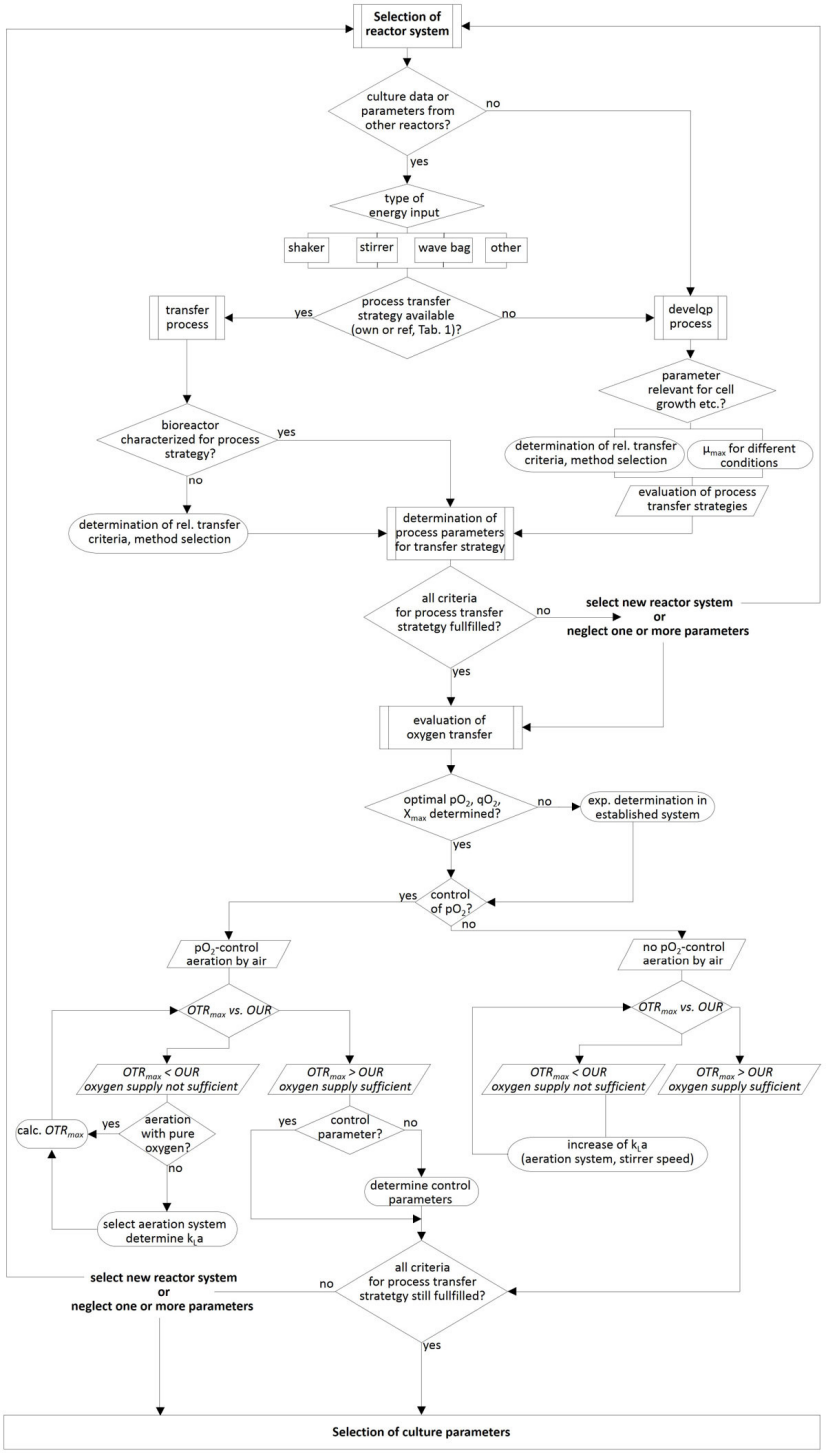
**Decision tree for selection of suitable cultivation parameters µ - growth rate, OTR - oxygen transfer rate, OUR - oxygen uptake rate, k_L_a - volume specific mass transfer coefficient**.

For selection and evaluation of suitable cultivation parameters a decision tree (Figure 1) has been formulated to provide a guideline for design of mammalian cell culture processes. References for process transfer strategies are given for the following cases:

• Scale similar and power imput similar: [1-3]

• Scale similar and power imput similar: [4-6]

• Scale up and power imput similar: [7,8]

• Scale up and power imput similar: [4,9,10]
